# Supplementary material for: Unique adipose tissue invariant natural killer T cell subpopulations control adipocyte turnover in mice
Source: Nat Commun. 2023 Dec 21;14:8512. doi: 10.1038/s41467-023-44181-3 (PMC10739728; doi:10.1038/s41467-023-44181-3)
Supplement: Supplementary file 1 — Supplementary Information [file 41467_2023_44181_MOESM1_ESM.pdf]

Supplementary Information

Unique adipose tissue invariant natural killer T cell subpopulations control adipocyte turnover in mice

Sang Mun Han, Eun Seo Park, Jeu Park, Hahn Nahmgoong,
Yoon Ha Choi, Jiyoung Oh, Kyung Min Yim, Won Taek Lee,
Yun Kyung Lee, Yong Geun Jeon, Kyung Cheul Shin, Jin Young Huh,
Sung Hee Choi, Jiyoung Park, Jong Kyoung Kim, and Jae Bum Kim

Supplementary Fig. 1

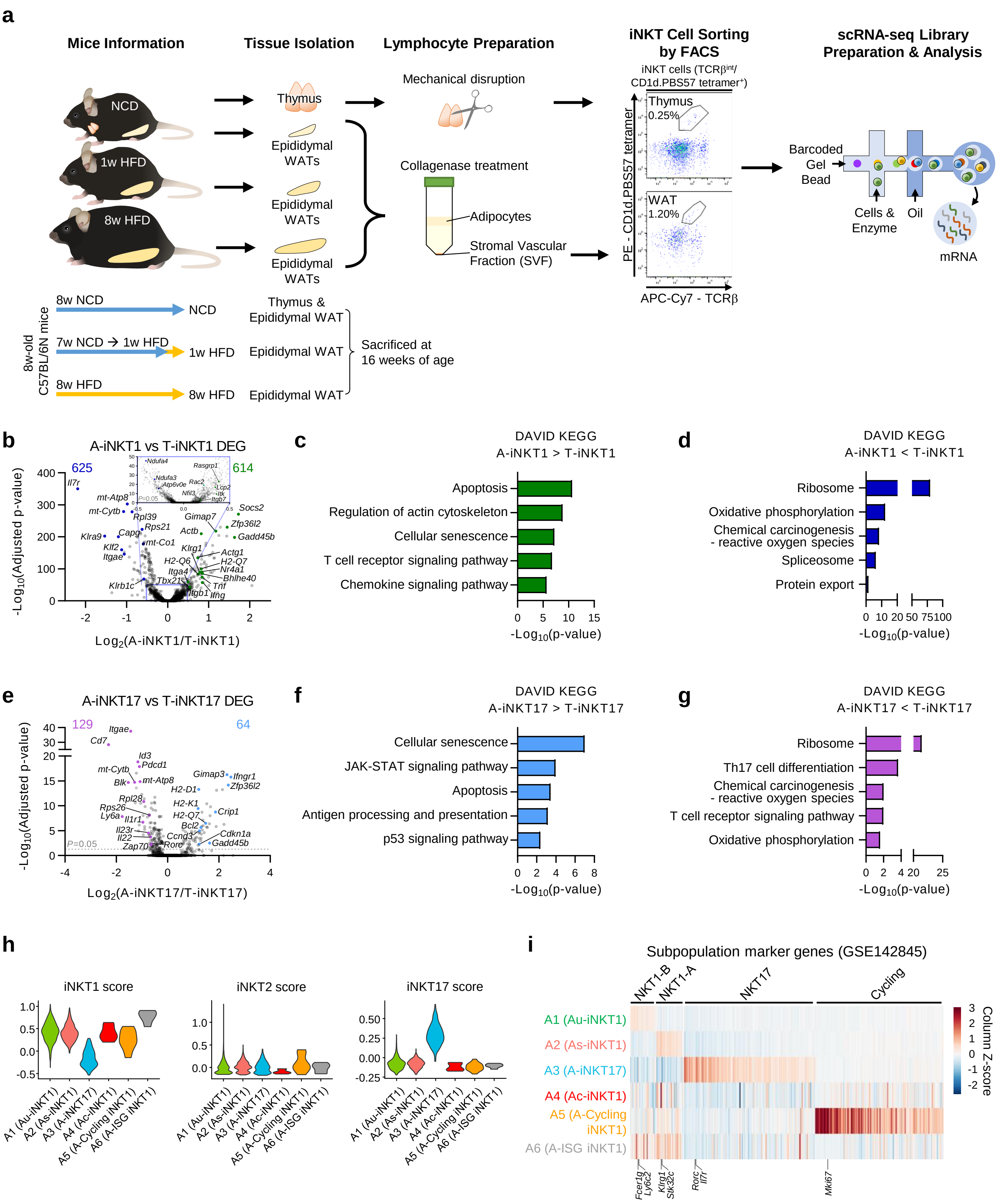

Supplementary Fig. 1 | Experimental scheme of scRNA-seq and analysis of differentially expressed genes between adipose iNKT cells and thymic iNKT cells. Related to Fig. 1.

a, Experimental scheme for scRNA-seq. Thymus and epididymal white adipose tissue (WAT) were extracted from 16-week-old male C57BL/6N mice. Stromal vascular fraction (SVF) was obtained through collagenase treatment. iNKT cells (TCRβ^{int}/CD1d.PBS57 tetramer^{+}) were obtained via flow cytometry. A scRNA-seq library was prepared using a 10X genomics platform. **b**, Differentially expressed genes (DEGs) between A-iNKT1 and T-iNKT1 cells (Adjusted P<0.05). **c**,**d**, KEGG pathway analysis of A-iNKT1 high-DEGs (**c**) and T-iNKT1 high-DEGs (**d**). **e**, DEGs between A-iNKT17 and T-iNKT17 cells (Adjusted P<0.05). **f**,**g**, KEGG pathway analysis of A-iNKT17 high-DEGs (**f**) and T-iNKT17 high-DEGs (**g**). DEGs are listed in Supplementary Data 1. **h**, Gene signature score of iNKT1, iNKT2, and iNKT17 in adipose iNKT cells. Used genes are listed in Supplementary Table 1. **i**, Heatmap showing the expression levels of subpopulation marker genes from previously reported adipose iNKT cell scRNA-seq data (GSE142845). Wilcoxon rank-sum test (two-tailed, adjusted for multiple comparisons using Bonferroni correction) (**b** and **e**). One-tailed Fisher's exact test (**c**, **d**, **f**, and **g**).

Supplementary Fig. 2

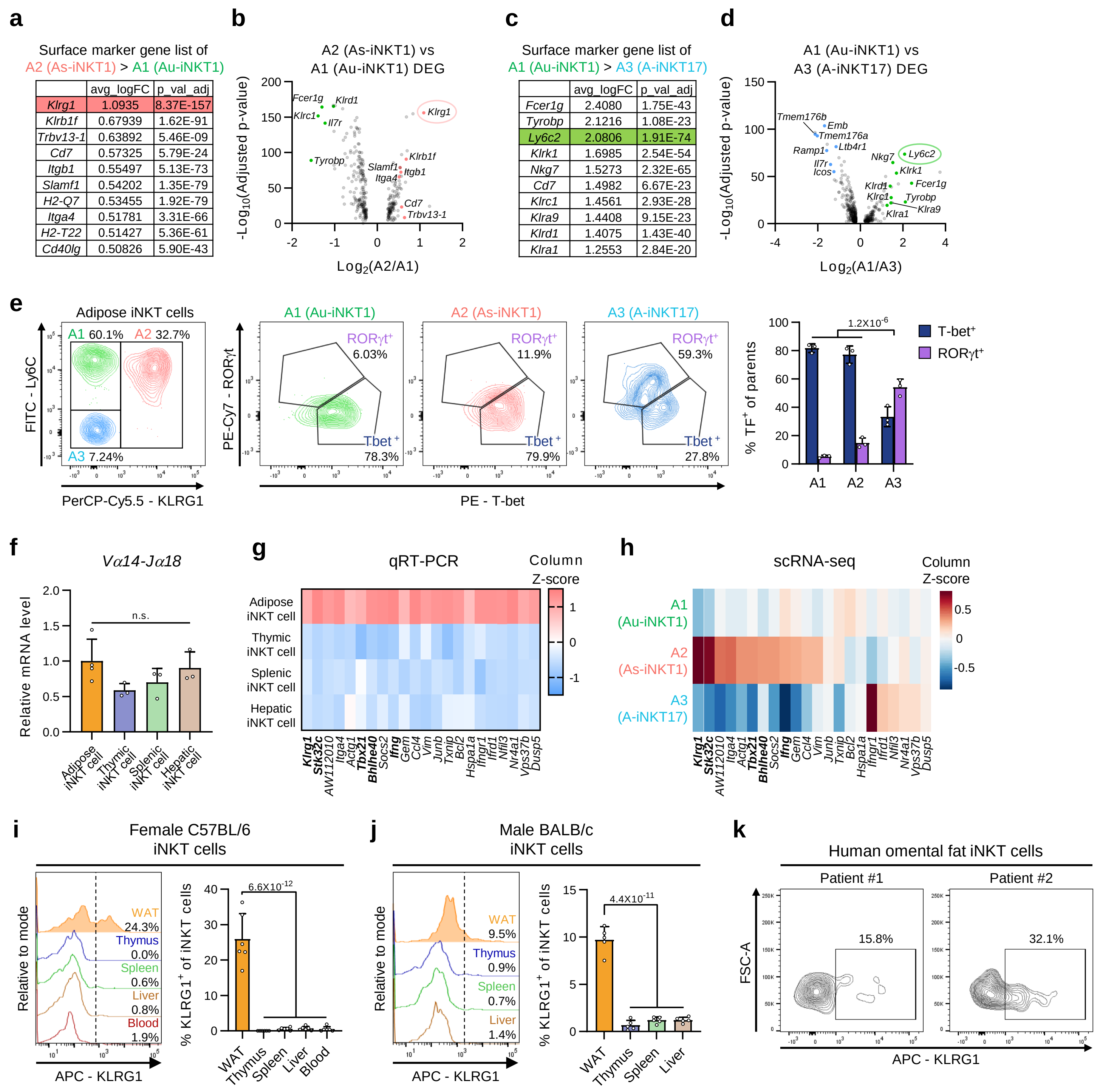

Supplementary Fig. 2 | Marker gene expression and validation of A1-A3 subpopulations. Related to Fig. 1.

a, Surface antigen gene list among A2 (As-iNKT1) subpopulation's upregulated differentially expressed genes (DEGs) compared to A1 (Au-iNKT1) subpopulation. **b**, Volcano plot of DEGs between A2 (As-iNKT1) and A1 (Au-iNKT1) subpopulation. **c**, Surface antigen gene list among A1 (Au-iNKT1) subpopulation's upregulated DEGs compared to A3 (A-iNKT17) subpopulation. **d**, Volcano plot of DEGs between A1 (Au-iNKT1) and A3 (A-iNKT17) subpopulation. **e**, Representative FACS plot and FACS-based proportion of T-bet and RORγt expression in each adipose iNKT cell subpopulation (n = 3). **f**, mRNA level of iNKT TCR alpha chain gene (*Vα14-Jα18*) in iNKT cells from WAT (n = 4), thymus (n = 3), spleen (n = 3), and liver (n = 3). **g**, Heatmap showing the mRNA levels of adipose iNKT cell-specific genes in iNKT cells from WAT (n = 4), thymus (n = 3), spleen (n = 3), and liver (n = 3). qRT-PCR data was converted to heatmap. **h**, Heatmap showing the expression levels of adipose iNKT cell-specific genes in A1 (Au-iNKT1), A2 (As-iNKT1), and A3 (A-iNKT17) subpopulations. **i**, Representative FACS plot and the proportion of $KLRG1^+$ iNKT cells from 10-week-old female C57BL/6 mice (n = 6). **j**, Representative FACS plot and the proportion of $KLRG1^+$ iNKT cells from 10-week-old male BALB/c mice (n = 5). **k**, Representative FACS plots of iNKT cells from human omental adipose tissue (n = 2). Data are represented as mean ± SD. n.s., non-significant. One-way ANOVA (**f**, **i**, and **j**). Two-way ANOVA (**e**). Wilcoxon rank-sum test (two-tailed, adjusted for multiple comparisons using Bonferroni correction) (**a**–**d**).

Supplementary Fig. 3

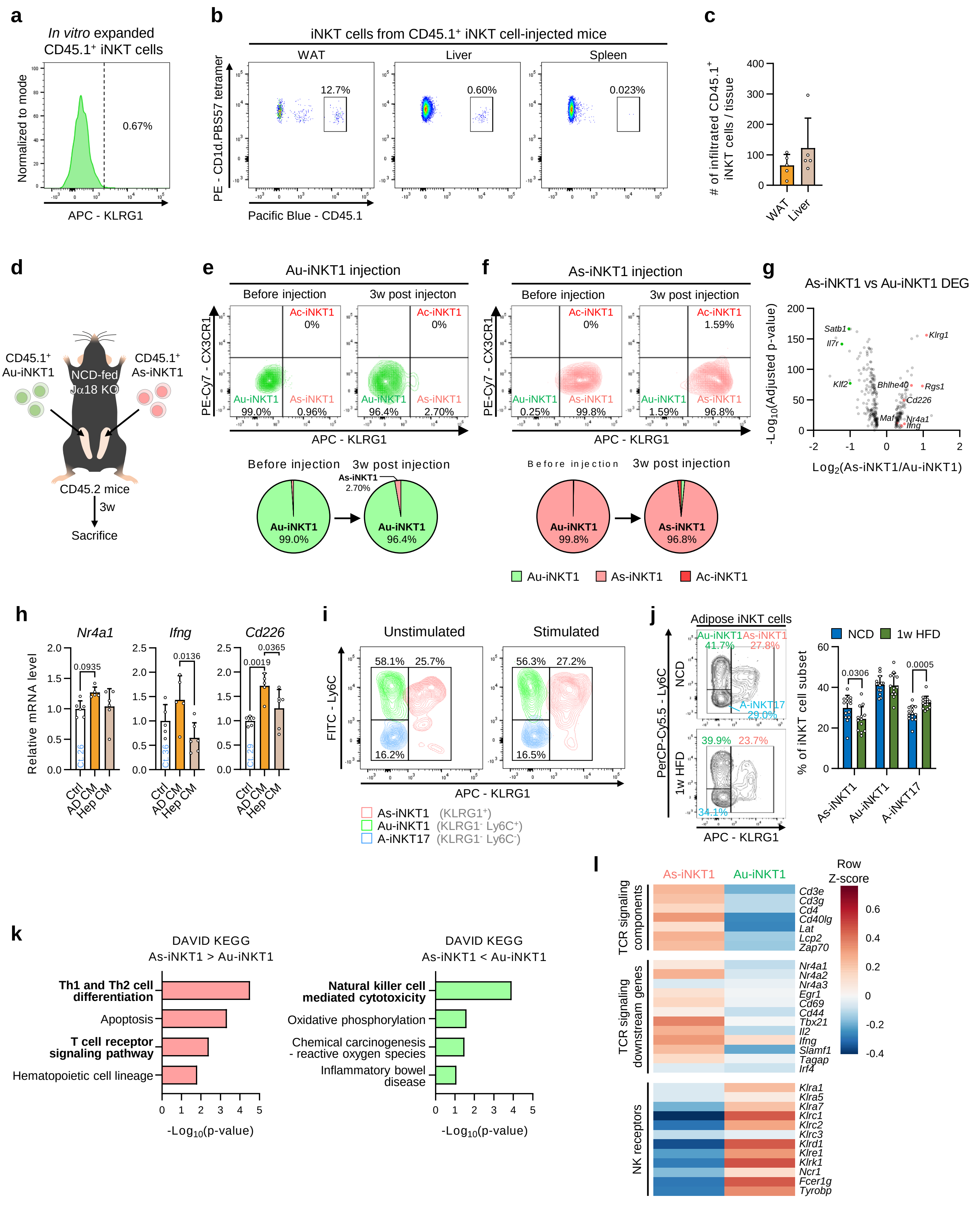

Supplementary Fig. 3 | Characterization of As-iNKT1 cells. Related to Fig. 2.

a, Representative FACS plot of KLRG1 expression in *in vitro* expanded primary $CD45.1^{+}$ iNKT cells. **b**, Representative FACS plots of $CD45.1^{+}$ iNKT cells infiltrated into WAT, liver, and spleen. 5×10^{5} of primary $CD45.1^{+}$ iNKT cells were intravenously injected into 3-week-old male mice and sacrificed 8 weeks after injection. **c**, Absolute number of infiltrated $CD45.1^{+}$ iNKT cells in WAT and liver per mouse in (**b**) (n = 5). **d**, Experimental scheme for adoptive transfer of $CD45.1^{+}$ Au-iNKT1 and As-iNKT1 cells. iNKT cells were sorted from CD45.1 mice 1-week after α-GC injection and injected into each WAT fat pad of 16-week-old CD45.2 Jα18 KO mice. **e**,**f**, FACS plots and composition of injected $CD45.1^{+}$ donor iNKT cells in recipient mice after 3 weeks (n = 1). **g**, Volcano plot of DEGs between As-iNKT1 and Au-iNKT1 cells. **h**, mRNA levels of *Nr4a1*, *Ifng*, and *Cd226* in DN32.D3 cells after 2 days of culture with Control media (Ctrl) (n = 6), WAT adipocyte-conditioned media (AD CM) (n = 5), or primary hepatocyte-conditioned media (Hep CM) (n = 6). **i**, Representative FACS plots of adipose iNKT cell subpopulations with or without activation using PMA/Ionomycin for 4 hours. **j**, Representative FACS plots and FACS-based proportion of each subpopulation among total adipose iNKT cells in NCD- (n = 14) or 1-week HFD-fed mice (n = 13). **k**, KEGG pathway analysis of As-iNKT1 high-DEGs and Au-iNKT1 high-DEGs ($P<0.05$). **l**, Heatmap showing the expression levels of TCR signaling pathway-related genes, TCR signaling downstream genes, and NK receptors. Data are represented as mean ± SD. One-way ANOVA (**h**). Two-tailed unpaired Student's t test (**j**). Wilcoxon rank-sum test (two-tailed, adjusted for multiple comparisons using Bonferroni correction) (**g**). One-tailed Fisher's exact test (**k**).

Supplementary Fig. 4

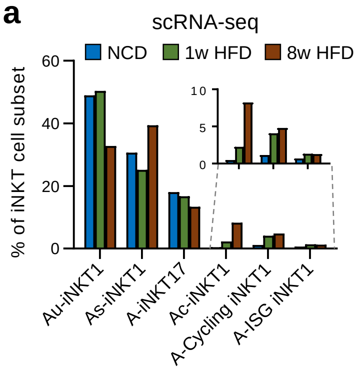

b

Surface marker gene list of
Ac-iNKT1 > As-iNKT1

	avg_logFC	p_val_adj
S1pr5	1.7507	9.49E-247
Cx3cr1	1.4182	6.31E-218
Klrd1	1.2655	7.83E-68
Klre1	1.0528	5.31E-60
Klrg1	0.98122	1.28E-53
Lgals1	0.91952	4.27E-34
Cd48	0.89631	1.75E-40
Il18rap	0.78509	1.42E-69
Ly6a2	0.75520	2.90E-62
Itga4	0.71982	1.16E-30

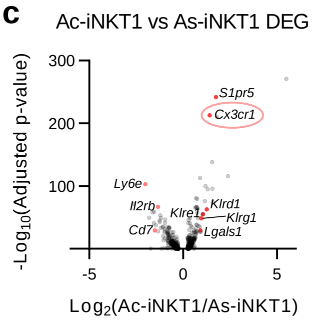

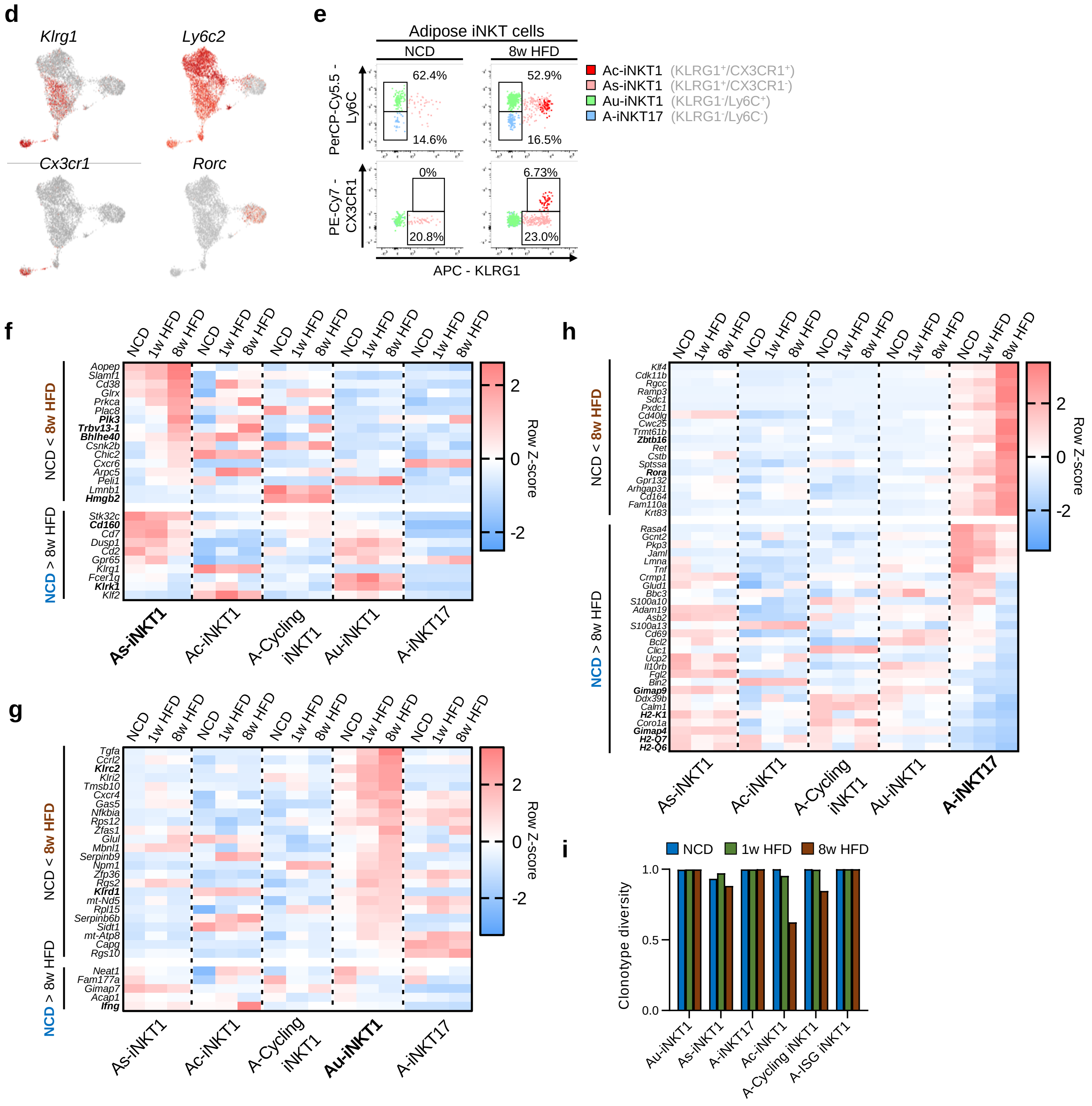

Supplementary Fig. 4 | Changes in adipose iNKT cells upon HFD-feeding. Related to Fig. 3.

a, Proportion of each adipose iNKT cell subpopulation in scRNA-seq data. **b**, Surface antigen gene list among Ac-iNKT1 cells' upregulated DEGs compared to As-iNKT1 cells. **c**, Volcano plot of DEGs between Ac-iNKT1 cells and As-iNKT1 cells. **d**, Gene expression levels of *Klrg1*, *Ly6c2*, *Cx3cr1*, and *Rorc* in adipose iNKT cells. **e**, Representative FACS plot of adipose iNKT cell subpopulations in NCD- or 8-week HFD-fed mice. **f**, Heatmap showing the expression levels of As-iNKT1-specifically changed genes upon 8-week HFD-feeding. **g**, Heatmap showing the expression levels of Au-iNKT1-specifically changed genes upon 8-week HFD-feeding. **h**, Heatmap showing the expression levels of A-iNKT17-specifically changed genes upon 8-week HFD-feeding. **i**, Clonotype diversity of each adipose iNKT cell subpopulation in NCD-, 1-week, or 8-week HFD-fed conditions. Wilcoxon rank-sum test (two-tailed, adjusted for multiple comparisons using Bonferroni correction) (**b** and **c**).

Supplementary Fig. 5

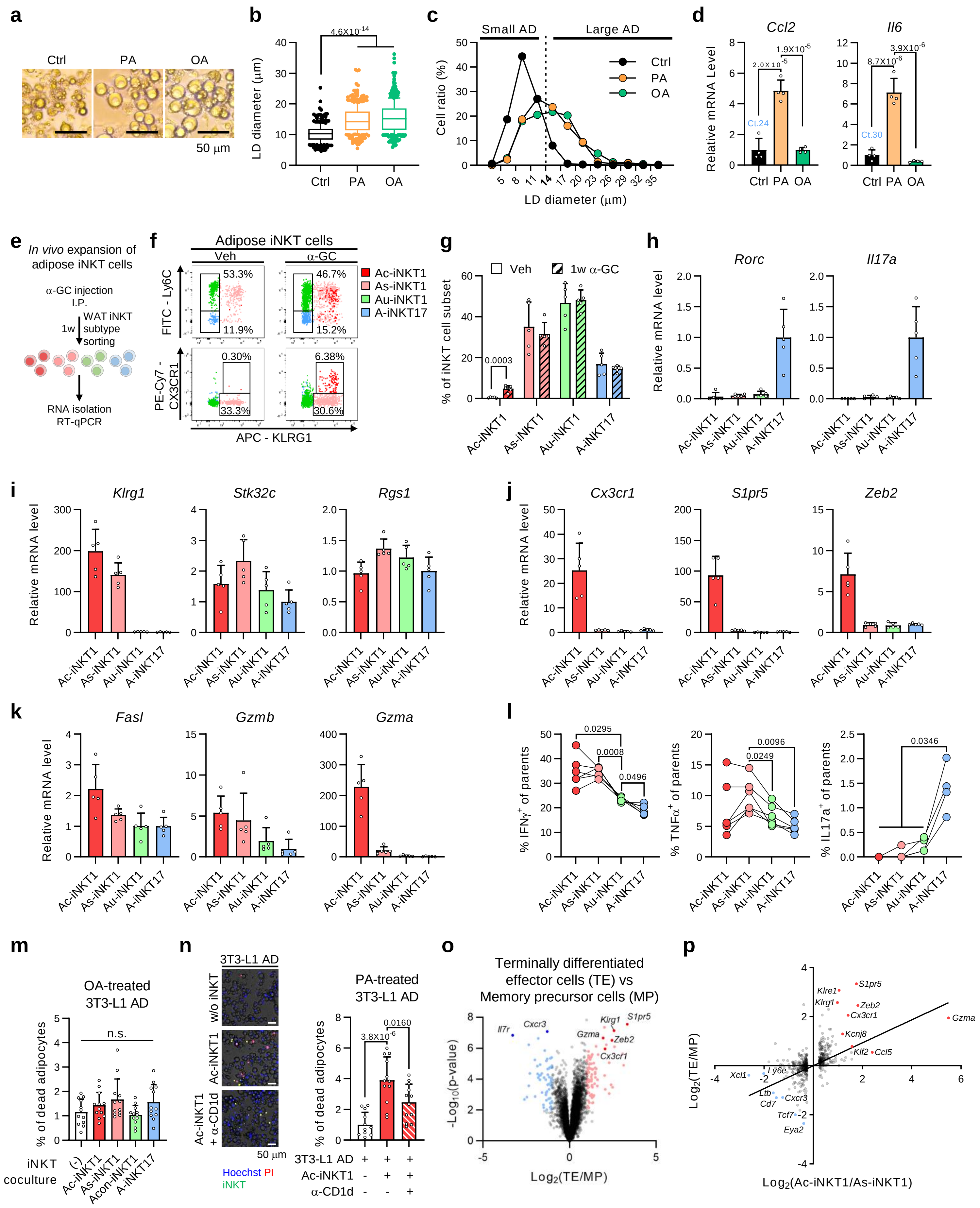

Supplementary Fig. 5 | *In vitro* adipocyte-iNKT cell coculture experiments and α-GC induced adipose iNKT cell expansion. Related to Fig. 4.

a, Representative images of 3T3-L1 adipocytes treated with Ctrl, palmitic acid (PA), or oleic acid (OA) media for 2 weeks. Scale bars, 50 µm. **b**,**c**, Lipid droplet (LD) diameters of Ctrl (n = 300), PA (n = 380), or OA media-treated 3T3-L1 adipocytes (n = 419) (**b**) and their distribution (**c**). **d**, mRNA expressions in Ctrl, PA, or OA media-treated 3T3-L1 adipocytes (n = 4). **e**, Experimental scheme for α-GC-induced *in vivo* adipose iNKT cell expansion. **f**,**g**, Representative FACS plots (**f**) and proportion (**g**) of adipose iNKT cell subpopulations in vehicle or α-GC injected mice (n = 5) in (**e**). **h**–**k**, mRNA expressions of A-iNKT17 cell marker genes (**h**), As-iNKT1 cell marker genes (**i**), Ac-iNKT1 marker genes (**j**), and cytotoxic marker genes (**k**) in adipose iNKT cell subpopulations after *in vivo* expansion (n = 5). **l**, Intracellular cytokine staining of *in vivo* expanded Ac-iNKT1, As-iNKT1, Au-iNKT1, and A-iNKT17 cells. Connected dots represent paired cell populations in a single SVF sample (IFNγ, TNFα (n = 5), and IL-17A (n = 4)). **m**, Proportion of PI^+ adipocytes among OA-treated 3T3-L1 adipocytes with or without adipose iNKT cell subpopulations (n = 12). **n**, Representative images and proportion of PI^+ adipocytes among PA-treated 3T3-L1 adipocytes with or without Ac-iNKT1 cells or CD1d neutralizing antibody (n = 12). Scale bars, 50 µm. **o**, Volcano plot of DEGs between TE and MP cells (GSE148681). **p**, Gene expression correlation between TE/MP signature and Ac-iNKT1/As-iNKT1 cell signature by using dataset in (**o**). TE/MP fold differences were calculated in DEGs between As-iNKT1 and Ac-iNKT1 cells. Data are represented as mean ± SD except (**b**) represented as box and whiskers plot. In (**b**), the lower, central, and upper line in each box represents the first (Q1), the second (median), and the third quartile (Q3), respectively. The whiskers extend from the box to the 10 and 90 percentile, respectively. n.s., non-significant. One-way ANOVA (**b**, **d**, **m**, and **n**). Paired one-way ANOVA (**l**). Two-tailed unpaired Student’s t test (**g** and **o**).

Supplementary Fig. 6

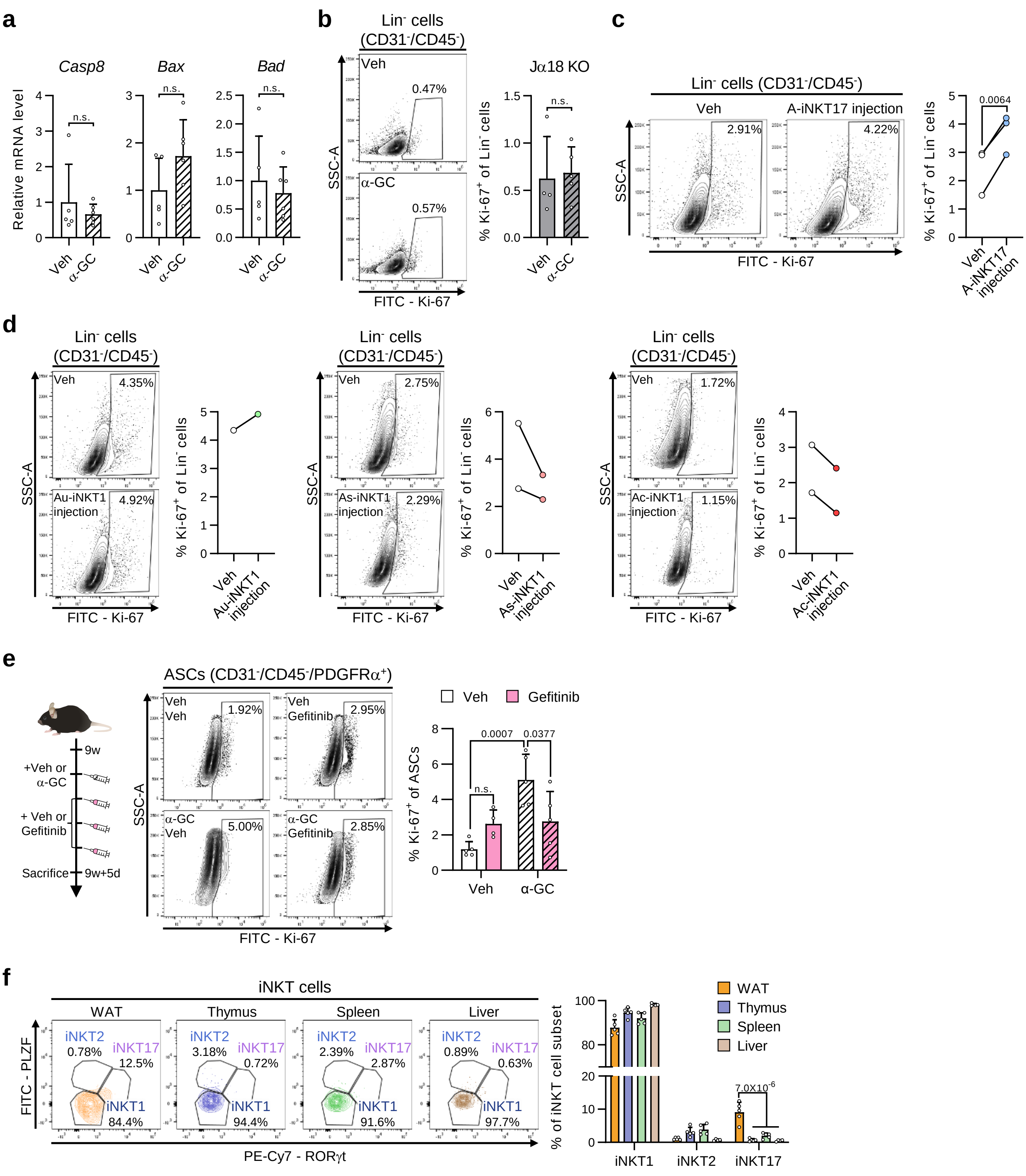

Supplementary Fig. 6 | iNKT cell activation does not induce apoptosis in NCD-fed mice and A-iNKT17, abundant in WAT, would stimulate ASC proliferation. Related to Fig. 5.

a, mRNA expressions of *Casp8*, *Bax*, and *Bad* in WAT from vehicle or α-GC injected mice. Mice were sacrificed 1 day after vehicle (n = 5) or α-GC injection (n = 6). **b**, Representative FACS plots and proportion of Ki-67$^+$ among lineage (Lin)-negative cells (CD31$^-$/CD45$^-$) from Jα18 KO WAT. Mice were sacrificed 4 days after vehicle (n = 4) or α-GC injection (n = 5). **c**, Representative FACS plots and the proportion of Ki-67$^+$ among WAT Lin-negative cells from 16-week HFD-fed Jα18 KO mice with or without A-iNKT17 cell injection (n = 3). Mice were sacrificed 3 weeks after injection. **d**, Representative FACS plots and proportion of Ki-67$^+$ among WAT Lin-negative cells from 16-week HFD-fed Jα18 KO mice with or without Au-iNKT1 (n = 1), As-iNKT1 (n = 2), or Ac-iNKT1 cell injection (n = 2). Mice were sacrificed 3 weeks after injection. **e**, Experimental scheme, representative FACS plots, and the ratio of proliferating ASCs (CD31$^-$/CD45$^-$/PDGFRα$^+$) in α-GC and/or Gefitinib, EGFR inhibitor injection (Veh/Veh (n = 5), Veh/Gefitinib (n = 4), α-GC/Veh (n = 5), and α-GC/Gefitinib (n = 5)). **f**, Representative FACS plots and the proportion of iNKT1, iNKT2, and iNKT17 cells from 10-week-old male C57BL/6 mice (WAT (n = 5), Thymus (n = 5), Spleen (n = 5), Liver (n = 4)). Data are represented as mean ± SD. n.s., non-significant. One-way ANOVA (**f**). Two-way ANOVA (**e**). Two-tailed unpaired Student's t test (**a** and **b**). Two-tailed paired student's t test (**c**).

Supplementary Fig. 7

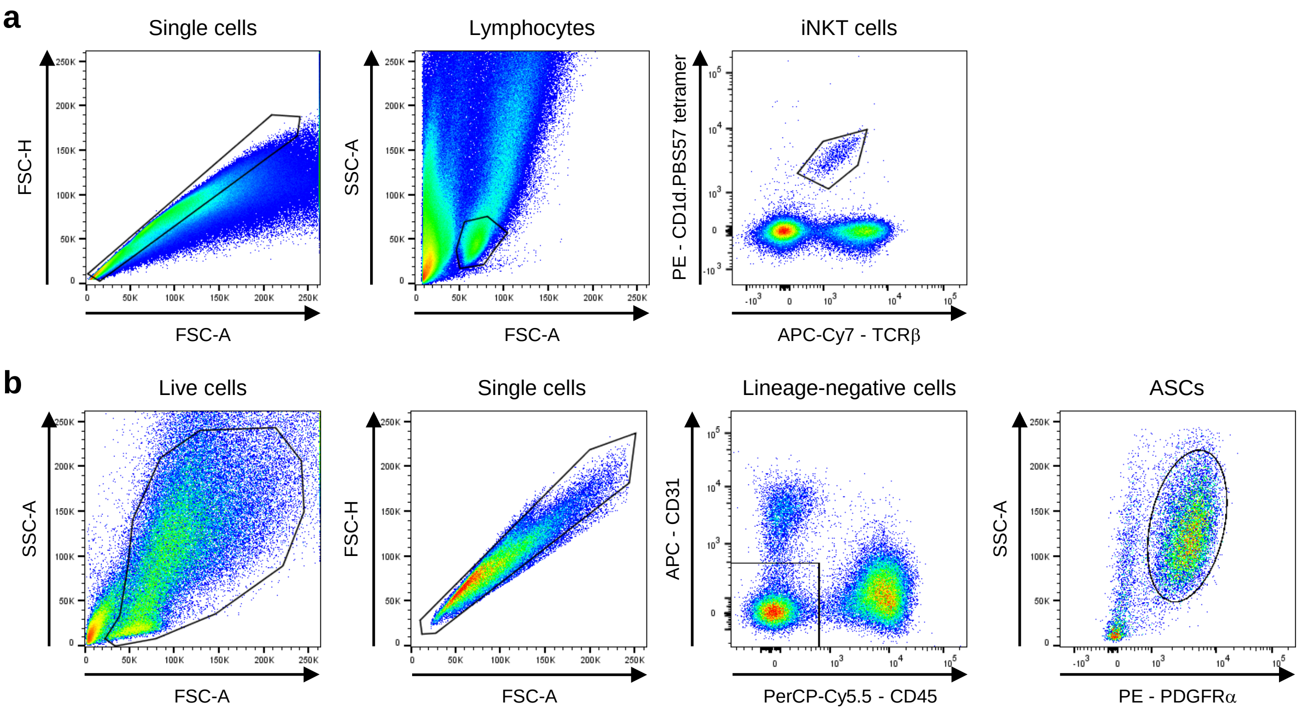

Supplementary Fig. 7 | Gating strategies for flow cytometry analysis of adipose iNKT cells and adipose stem cells.

a, Representative FACS plots to identify iNKT cells from WAT. **b**, Representative FACS plots to identify ASCs from WAT.

Supplementary Table 1

Gene List		
iNKT1 score	iNKT2 score	iNKT17 score
Nkg7	*Plac8*	*Tmem176a*
Tbx21	*Tesc*	*Serpinb1a*
Klrd1	*9530036M11Rik*	*Blk*
Ifitm10	*Il4*	*Tmem176b*
Ly6c2	*Psmg2*	*Pxdc1*
Klrb1c	*Zbtb16*	*S100a4*
Klrk1	*Izumo1r*	*Actn2*
Xcl1	*Drosha*	*Il1r1*
Fasl	*Slamf6*	*Il23r*
Gzmb		*Ccr6*
Cxcr3		*Rorc*
Gimap3		*Aqp3*
Klra3		*Lrrc17*
Ms4a4b		*Tuba8*
Klra9		*Il17re*
AW112010		*Cabin1*
Slamf7		*Cd7*
Klrc2		*Chad*
Lrrk1		*Apol7b*
H2-Q7		*Tnfrsf25*
Il2rb		*Stab2*
Rgs1		*Emb*
Fcer1g		*Sdc1*
Hsd11b1		*Itgae*
H2-Q6		*Kcnk1*
Stat4		*Cxcr6*
Fgl2		*Avpi1*
Klre1		*Prelid2*
Ctla2a		*Ramp3*
Klra5		*5830411N06Rik*
Klrc1		*Plekhf1*
Dapk2		*Abhd15*
Ppm1j		*Abi3bp*
Styk1		*Jag1*
Il12rb2		*Mycn*
Itga1		*Vax2*
H2-K1		*Npl*
Pik3ap1		*1700113H08Rik*
Klra8		*Gm16271*
Klra		*Mmp25*
Klra10		*Rnf208*

Supplementary Table 2

Primers for qRT-PCR			
Species	Gene	Forward (5' to 3')	Reverse (5' to 3')
Mouse	*Actg1*	CCCTATCGAACACGGCATTG	CCTGAATGGCCACGTACATG
	Adipoq	GGCAGGAAAGGAGAGCCTGG	GGCCTTGTCCTTCTTGAAGA
	Areg	GCAGATACATCGAGAACCTGGAG	CCTTGTCATCCTCGCTGTGAGT
	AW112010	GATGCAACAATACCTGGCGT	TGACGACCTGGGTCTGGTAT
	Bad	GGGATGGAGGAGGAGCTTAG	CCCACCAGGACTGGATAATG
	Bax	TGGAGATGAACTGGACAGCA	GATCAGCTCGGGCACTTTAG
	Bcl2	AGGAGCAGGTGCCTACAAGA	GCATTTTCCCACCACTGTCT
	Bhlhe40	TGGTGATTTGTCGGGAAGAAA	ACGGGCACAAGTCTGGAAAC
	Casp8	ATCCTATCCCACGGTGACAA	TGTGGTTCTGTTGCTCGAAG
	Ccl2	AGGTCCCTGTCATGCTTCTG	TCTGGACCCATTCCTTCTTG
	Ccl4	TTCCTGCTGTTTCTCTTACACCT	CTGTCTGCCTCTTTTGGTCAG
	Ccl5	GCTGCTTTGCCTACCTCTCC	TCGAGTGACAAACACGACTGC
	Cd1d1	ACGTCCTGGCAGACAGTCCCAGG	TTAATGTTGAAAAGAGCGTACTGGC
	Cd226	CTGTCTGCAGAACCTGGACA	CATGGCATTTGGAATGATGA
	Cx3cr1	CAGCATCGACCGGTACCTT	GCTGCACTGTCCGGTTGTT
	Dusp5	ACCACCCACCTACACTACAA	CCTTCTTCCCTGACACAGTCAATA
	Fasl	CCCCAGTACACCCTCTGAAA	CAAGACTGACCCCGGAAGTA
	Gem	ACAGCGACTGTGAGGTCTTG	GCCATTCGTTCTCCCCCTTA
	Gzma	TGTGAAACCAGGAACCAGATG	GGTGATGCCTCGCAAAATA
	Gzmb	TCGACCCTACATGGCCTTAC	TGGGGAATGCATTTTACCAT
	Hspa1a	TGGTGCAGTCCGACATGAAG	GCTGAGAGTCGTTGAAGTAGGC
	Ifng	TACTGCCACGGCACAGTCATTGAA	GCAGCGACTCCTTTTCCGCTTCCT
	Ifngr1	AGGTGTATTCGGGTTCCTGG	AATACGAGGACGGAGAGCTG
	Ifrd1	AGAGTGCGAAGACAAGACAGG	TTCAGACAGCGCTCAATGCT
	Il17a	TCCAGAAGGCCCTCAGACTA	AGCATCTTCTCGACCCTGAA
	Il6	AGTTGCCTTCTTGGGACTGA	TCCACGATTTCCCAGAGAAC
	Il7r	GGAAGTGGATGGAAGTCAAC	TGCGATAAACGACTTTCAGGT
	Itga4	CTCCCTCAAGATGATAAGTTGTTCAA	TGTGCAAATGTACACTCTCTTCCA
	Junb	GACCTGCACAAGATGAACCACG	ACTGCTGAGGTTGGTGTAGACG
	Klf2	TGTGAGAAATGCCTTTGAGTTTACTG	CCCTTATAGAAATACAATCGGTCATAGTC
	Klrg1	CCTCTGGACGAGGAATGGTA	ACCTCCAGCCATCAATGTTC
	Maf	AGCAGTTGGTGACCATGTCG	TGGAGATCTCCTGCTTGAGG
	Nfil3	CAGTGCAGGTGACGAACATT	TTCCACCACACCTGTTTTGA
	Nr4a1	TGTGAGGGCTGCAAGGGCTTC	AAGCGGCAGAACTGGCAGCGG
	Pnpla2	ACCATCACAGTGTCCCCATT	CTCCAGCGGCAGAGTATAGG
	Rgs1	TTGGAATGGACGTGAAAACA	CCTCACAAGCCAACCAGAAT
	Rorc	TGCAAGACTCATCGACAAGG	AGGGGATTCAACATCAGTGC
	Rplp0 (Normalization gene)	GAGGAATCAGATGAGGATATGGGA	AAGCAGGCTGACTTGGTTGC
	S1pr5	GATCCCTTCCTGGGTCTAGC	TAGAGCTGCGATCCAAGGTT
	Satb1	TGATAGAGATGGCGTTGCTG	TTTTGAGGGTGACCACATGA
	Socs2	CTGCGCGAGCTCAGTCAAA	CAATCCGCAGGTTAGTCGGT
	Stk32c	CCTTTGAGCTGGAGGAGATG	TCACGAAGTCTTGCTGGATG
	Tbx21	AGCAAGGACGGCGAATGTT	GGGTGGACATATAAGCGGTTC
	Txnip	ATCCCAGATACCCCAGAAGC	TGAGAGTCGTCCACATCGTC
	Va14-Ja18	CTAAGCACAGCACGCTGCAC	CAGGTATGACAATCAGCTGACTCC
	Vim	CCTGGCCGAGGACATCAT	TTCAAGGTCAAGACGTGCCA
	Vps37b	AGGACACTGAGAACATGGCAG	TCCGCTTGCTCTGGTAGAC
	Zeb2	CATGAACCCATTTAGTGCCA	AGCAAGTCTCCCTGAAATCC
