# Supplementary material for: Unique adipose tissue invariant natural killer T cell subpopulations control adipocyte turnover in mice
Source: Nat Commun. 2023 Dec 21;14:8512. doi: 10.1038/s41467-023-44181-3 (PMC10739728; doi:10.1038/s41467-023-44181-3)
Supplement: Supplementary file 3 — Description of additional supplementary files [file 41467_2023_44181_MOESM3_ESM.docx]

**Supplementary Data 1 | List of differentially expressed genes between adipose and thymic iNKT cells**

Differentially expressed genes between adipose iNKT1 cells and thymic iNKT1 cells, and adipose iNKT17 cells and thymic iNKT17 cells from NCD-fed mice (adjusted *P*<0.05). P-values were calculated by using two-tailed Wilcoxon rank-sum test and adjusted for multiple comparisons using Bonferroni correction.
